# Novel facultative Methylocella strains are active methane consumers at terrestrial natural gas seeps

**DOI:** 10.1186/s40168-019-0741-3

**Published:** 2019-10-04

**Authors:** Muhammad Farhan Ul Haque, Andrew T. Crombie, J. Colin Murrell

**Affiliations:** 1 School of Environmental Sciences, University of East Anglia, Norwich, NR4 7TJ UK; 2 School of Biological Sciences, University of the Punjab, Lahore, Pakistan; 3 School of Biological Sciences, University of East Anglia, Norwich, NR4 7TJ UK

**Keywords:** *Methylocella*, Facultative methanotrophs, Propanotrophs, Natural gas, Biological methane, Geological methane, DNA stable isotope probing, Soluble methane monooxygenase, XoxF-methanol dehydrogenase

## Abstract

**Background:**

Natural gas seeps contribute to global climate change by releasing substantial amounts of the potent greenhouse gas methane and other climate-active gases including ethane and propane to the atmosphere. However, methanotrophs, bacteria capable of utilising methane as the sole source of carbon and energy, play a significant role in reducing the emissions of methane from many environments. *Methylocella*-like facultative methanotrophs are a unique group of bacteria that grow on other components of natural gas (i.e. ethane and propane) in addition to methane but a little is known about the distribution and activity of *Methylocella* in the environment. The purposes of this study were to identify bacteria involved in cycling methane emitted from natural gas seeps and, most importantly, to investigate if *Methylocella*-like facultative methanotrophs were active utilisers of natural gas at seep sites.

**Results:**

The community structure of active methane-consuming bacteria in samples from natural gas seeps from Andreiasu Everlasting Fire (Romania) and Pipe Creek (NY, USA) was investigated by DNA stable isotope probing (DNA-SIP) using ^13^C-labelled methane. The 16S rRNA gene sequences retrieved from DNA-SIP experiments revealed that of various active methanotrophs, *Methylocella* was the only active methanotrophic genus common to both natural gas seep environments. We also isolated novel facultative methanotrophs, *Methylocella* sp. PC1 and PC4 from Pipe Creek, able to utilise methane, ethane, propane and various non-gaseous multicarbon compounds. Functional and comparative genomics of these new isolates revealed genomic and physiological divergence from already known methanotrophs, in particular, the absence of *mxa* genes encoding calcium-containing methanol dehydrogenase. *Methylocella* sp. PC1 and PC4 had only the soluble methane monooxygenase (sMMO) and lanthanide-dependent methanol dehydrogenase (XoxF). These are the first *Alphaproteobacteria* methanotrophs discovered with this reduced functional redundancy for C-1 metabolism (i.e. sMMO only and XoxF only).

**Conclusions:**

Here, we provide evidence, using culture-dependent and culture-independent methods, that *Methylocella* are abundant and active at terrestrial natural gas seeps, suggesting that they play a significant role in the biogeochemical cycling of these gaseous alkanes. This might also be significant for the design of biotechnological strategies for controlling natural gas emissions, which are increasing globally due to unconventional exploitation of oil and gas.

**Electronic supplementary material:**

The online version of this article (10.1186/s40168-019-0741-3) contains supplementary material, which is available to authorized users.

## Background

Methane, a potent greenhouse gas, is one of the most significant contributors to climate change. Emissions since the Industrial Revolution have driven a large increase in the atmospheric concentrations of methane, currently around 1.8 ppm, an increase by a factor of 2.6 from pre-industrial times [www.esrl.noaa.gov/gmd/ccgg/trends_ch4/]. Globally, approximately 600–900 Tg of methane is emitted annually from various natural and anthropogenic sources [1]. Based on the process of methane synthesis, it can be categorised as arising from two origins. Firstly, biogenic methane is produced by methanogenic archaea, under anaerobic conditions, mainly in wetlands, landfill sites, rice paddies, the rumen of cattle and the hindgut of termites. Secondly, methane is produced from the thermogenic decay of sedimentary organic material, resulting in a mixture of methane and other gases commonly known as natural gas. The origin of methane-rich gas can be determined by its chemical composition and by measurement of the stable isotopic ratios of carbon (C) and hydrogen [2, 3].

Natural gas is emitted to the atmosphere from subsurface reservoirs, through natural seepage or during mining and extraction of coal and hydrocarbons. These sources contribute a significant amount of methane (42–64 Tg year^−1^) and other climate-active gases, e.g. ethane (a photochemical pollutant, 2–4 Tg year^−1^) and propane (an ozone precursor, 1–2.4 Tg year^−1^) [4, 5]. Seepage of natural gas occurs over a wide range of terrestrial hydrocarbon-prone sedimentary basins, both as visible features including dry gas seeps and mud volcanoes, hot and cold springs, alkaline soda lakes and volcanic systems [2, 6–11] and also as diffused and frequently undetected microseepage [12]. In marine environments, natural gas is emitted from deep sea hydrothermal vents and shallow marine methane seeps [13, 14]. Terrestrial natural gas seeps have been reported for centuries in many regions, including the Appalachian Basin in the USA [15–17], represented by towns such as Gasport (Niagara County, USA), thus named in 1826. Many of these seeps emit natural gas, which can be ignited [18]. Recently a seep, reputedly known to Native Americans thousands of years ago, named the “Eternal Flame” (Chestnut Ridge National Park USA) was highlighted, where remarkable releases of natural gas were observed [19]. The gas released from this site contains methane (60%, v/v) plus ethane (23%, v/v) and propane (12%, v/v) [19]. Human activities have also caused major natural gas releases, e.g. the Deepwater Horizon disaster of 2010 released 170,000 t of natural gas [20]. Geological events such as thawing glaciers [21], as well as the exploitation of unconventional oil and gas reserves, including shale gas extraction (fracking) are predicted to increase the release of geological methane, with accompanying concerns of environmental pollution and climate change [22–25].

However, methanotrophic bacteria, a unique group of microbes that utilise methane as their sole source of C and energy, can consume methane before it reaches the atmosphere and have been reported to mop up over half of the methane produced by methanogens in wetlands [26–28]. Phylogenetically, most aerobic methanotrophs belong to the phyla *Proteobacteria* (*Alphaproteobacteria*, *Gammaproteobacteria*) and *Verrucomicrobia* (although *Verrucomicrobia* comprises both methanotrophs and non-methanotrophs, preventing designation as methanotrophs by taxonomy alone in this case). They contain the enzyme methane monooxygenase (MMO) which catalyses the oxidation of methane to methanol [29]. There are two types of MMO: a copper-containing, membrane-bound, particulate methane monooxygenase (pMMO) and a diiron centre-containing, cytoplasmic, soluble methane monooxygenase (sMMO) [29–32]. After the initial oxidation of methane to methanol, methanol dehydrogenase oxidises methanol to formaldehyde, which can be assimilated to cell carbon or further oxidised to formate and CO_2_ for energy generation [31]. There are two types of methanol dehydrogenase common in methanotrophs; a calcium-containing enzyme encoded by the *mxa* gene cluster and a lanthanide-containing variant (XoxF) encoded by *xoxF* [33–35]. Although *xoxF* genes were detected in methanotrophs many years ago, their function was not established until the discovery of the role of lanthanides (rare earth elements) as co-factors [36]. Recently, several studies have shown that lanthanides regulate the expression of both types of methanol dehydrogenases in methanotrophs [37–42] and methylotrophs [43–46]. These findings confirm that lanthanide-containing XoxF is also environmentally important, in addition to the calcium-containing methanol dehydrogenase [35, 47].

Methanotrophs were considered obligate for decades until the discovery of *Methylocella*, a facultative genus capable of growing on several multicarbon compounds in addition to methane [48, 49]. *Methylocella* belong to the family *Beijerinckiaceae* (*Alphaproteobacteria*) that comprises diversified heterotrophs ranging from generalist organotrophs (e.g. *Beijerinckia indica*) to facultative methanotrophs (e.g. *Methylocella silvestris*) and obligate methanotrophs (e.g. *Methylocapsa acidiphilia*) [50]. Recently, some more limited facultative methanotrophs of the genera *Methylocystis* and *Methylocapsa*, which can grow on ethanol or acetate in addition to C-1 substrates, have been described [51–54]. Analysis of the genome of *Methylocella silvestris* BL2 revealed that, unlike most methanotrophs, *Methylocella* uses only the sMMO to oxidise methane and does not contain the pMMO [55]. For decades, ecological studies, which often rely on the pMMO gene markers, have identified obligate methanotrophs as prevalent in environments rich in methane emissions [56, 57] and with the exception of a few previous studies [58, 59] little is known about the distribution of *Methylocella* in the environment.

Microbes growing on other components of natural gas, such as ethane and propane, include mainly *Actinobacteria* (e.g. *Rhodococcus*, *Nocardioides* and *Mycobacterium*) [60–63], *Gammaproteobacteria* (*Pseudomonas*) [64] or *Betaproteobacteria* (*Thauera*) [65, 66]. Many propanotrophs contain a propane monooxygenase (PrMO) enabling growth on ethane and propane [67, 68]. Propanotrophs are metabolically versatile compared to methanotrophs, and grow on a range of multicarbon compounds, but not methane [67]. PrMO of propanotrophs and sMMO of methanotrophs form two distinct groups within a large family of enzymes known as soluble di-iron monooxygenases (SDIMOs) [69–71].

*Methylocella silvestris* BL2 also contains a PrMO and can grow on methane and propane simultaneously using sMMO and PrMO [49]. The metabolic versatility of *Methylocella* to utilise several components of natural gas is unique and suggests a potentially significant role for *Methylocella*-like facultative methanotrophs in the biogeochemical cycling of natural gas in the environment. We hypothesised that less versatile obligate methanotrophs and propanotrophs at natural gas seep sites may be at a competitive disadvantage compared to *Methylocella*. Terrestrial natural gas seeps have been largely ignored in the past in terms of methanotrophic studies [72–74] compared to the studies targeting marine hydrocarbons seeps [75–81]. Recently, we reported that *Methylocella*-like facultative methanotrophs are abundant at natural gas seep sites [59]. However, these data did not reveal the activity of abundant taxa. Therefore, the aim of this study was to identify the active microbes involved in cycling natural gas methane and determine if *Methylocella*-like facultative methanotrophs are active at terrestrial seep sites, using both culture-dependent and culture-independent methods.

## Results and discussion

### Active methanotrophs at terrestrial natural gas seeps revealed by DNA stable isotope probing (DNA-SIP)

**Fig. 1:**
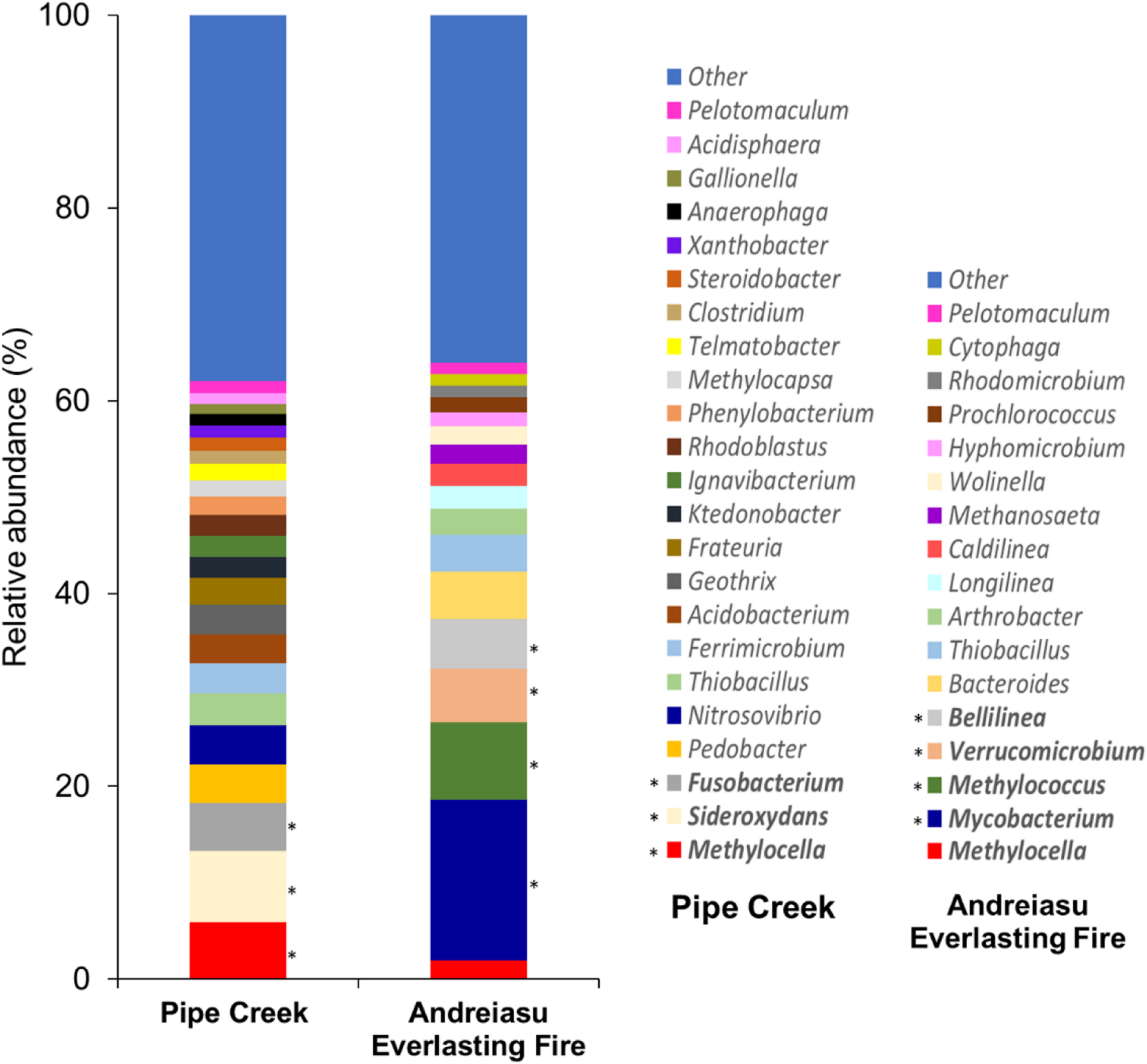
Relative abundance of bacterial genera in native environmental samples from Pipe Creek and Andreiasu Everlasting Fire, based on 16S rRNA gene amplicon sequencing. Genera with a relative abundance of higher than 5% are shown in bold and with an asterisk (*). 16S rRNA gene amplicon sequence data for Andreiasu Everlasting Fire were reported in Farhan Ul Haque et al. [59]

We sampled two different natural gas seeps reported to emit thermogenic natural gas containing methane, ethane and propane; Andreiasu Everlasting Fire, Romania, with a slightly basic pH (pH 8.2), and Pipe Creek, New York, USA, with slightly acidic pH (pH 6.0) [7, 18, 59]. Amplicon sequencing targeting the 16S rRNA gene in DNA samples isolated directly from these environments was performed to investigate the native bacterial community of the seep sites. Sequence analysis showed that out of 12 phyla at an abundance of higher than 1%, *Proteobacteria* (alpha, beta and gamma), *Actinobacteria*, *Bacteroidetes* and *Chloroflexi* formed the major part (> 70%) of native bacterial communities in unenriched environmental samples (Additional file 1: Figure S1). Dominant taxa included *Sideroxydans* (Pipe Creek) and *Mycobacterium* (Andreiasu Everlasting Fire) when analysed at the genus level (Fig. 1). As reported previously [59], among methanotrophs, *Methylocella* and *Methylocapsa* dominated in samples from Pipe Creek while *Methylococcus* and *Methylocella* were the most abundant methanotrophs in Andreiasu Everlasting Fire (Fig. 1). *Verrucomicrobia* were also abundant in samples from Andreiasu Everlasting Fire, but cannot be definitively identified as methanotrophs based on 16S rRNA gene phylogeny. Comparatively, the relative abundance of *Methylocella* was higher at Pipe Creek (5.92% ± 0.1) than at Andreiasu Everlasting Fire (1.89% ± 0.01). Interestingly, the proportion of ethane and propane in the natural gas released from the Pipe Creek seep (22% v/v, ethane and propane) was also several times higher than in the gas released from Andreiasu Everlasting Fire (3% v/v, ethane and propane) [7, 18, 59]. This suggested that increased ethane and propane content of natural gas might favour *Methylocella*-like facultative methanotrophs to colonise natural gas seep sites [59, 82].

**Fig. 2:**
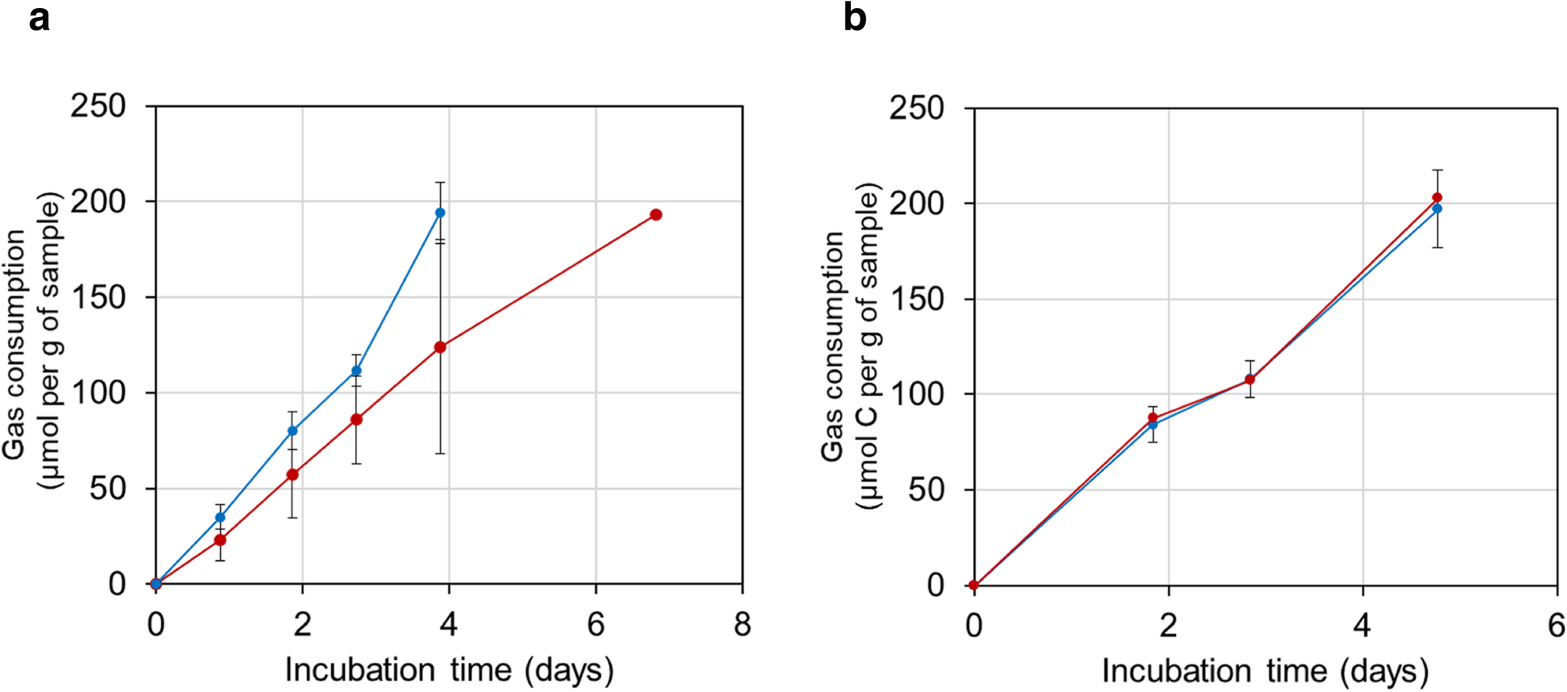
Consumption of methane by environmental samples from natural gas seep sites of Pipe Creek (**a**) and Andreiasu Everlasting Fire (**b**). Microcosms containing environmental samples were incubated under ^13^C-methane (red circles) or ^12^C-methane (blue circles) as the only sources of C or energy without any supplementary nutrients. The total amount of methane was injected into the headspace in two spikes (approximately 100 μmol/gram of fresh sample per spike). Data points show the mean (with error bars showing the standard errors) of duplicate incubations for each substrate

The community structure of active methane-consuming bacteria in samples from natural gas seep sites was investigated by DNA-SIP [83], using ^13^C-methane incubations in parallel with ^12^C-methane incubations (as control) at two time points (determined by incorporation of total methane consumed, i.e. 100 and 200 μmol methane utilised per gram sample). Rates of consumption of ^13^C-methane or ^12^C-methane observed in these samples from natural gas seeps (Fig. 2, Table 1) were faster than those reported for biogenic methane consumption in agricultural wetlands [84, 85]. This suggests that these natural gas seep sites may be hotspots for methane oxidation and potentially a large active biological sink for methane. DNA recovered from CsCl fractions of SIP incubations revealed that 200 μmol methane utilised per gram of sample (approximately 100 μmol C assimilated to biomass per gram of sample) resulted in the incorporation of sufficient ^13^C label for a successful DNA-SIP experiment (Additional file 1: Figure S2).

**Table 1:** Potential rates^1,2^ of methane utilisation by environmental samples

Substrate gas	Pipe Creek	Andreiasu Everlasting Fire
^13^C-methane	34.41 (± 9.53)	38.33 (± 0.16)
^12^C-methane	44.84 (± 3.39)	38.25 (± 3.58)

^1^Values (μmol methane per gram of fresh sample per day) are calculated based on the methane consumed by fresh environmental samples incubated in lab scale microcosms (120 ml sealed serum vials) with ^13^C-methane or ^12^C-methane injected into the headspace (1%, v/v)

^2^Average values of biological duplicates (± standard error of means) are presented

**Fig. 3:**
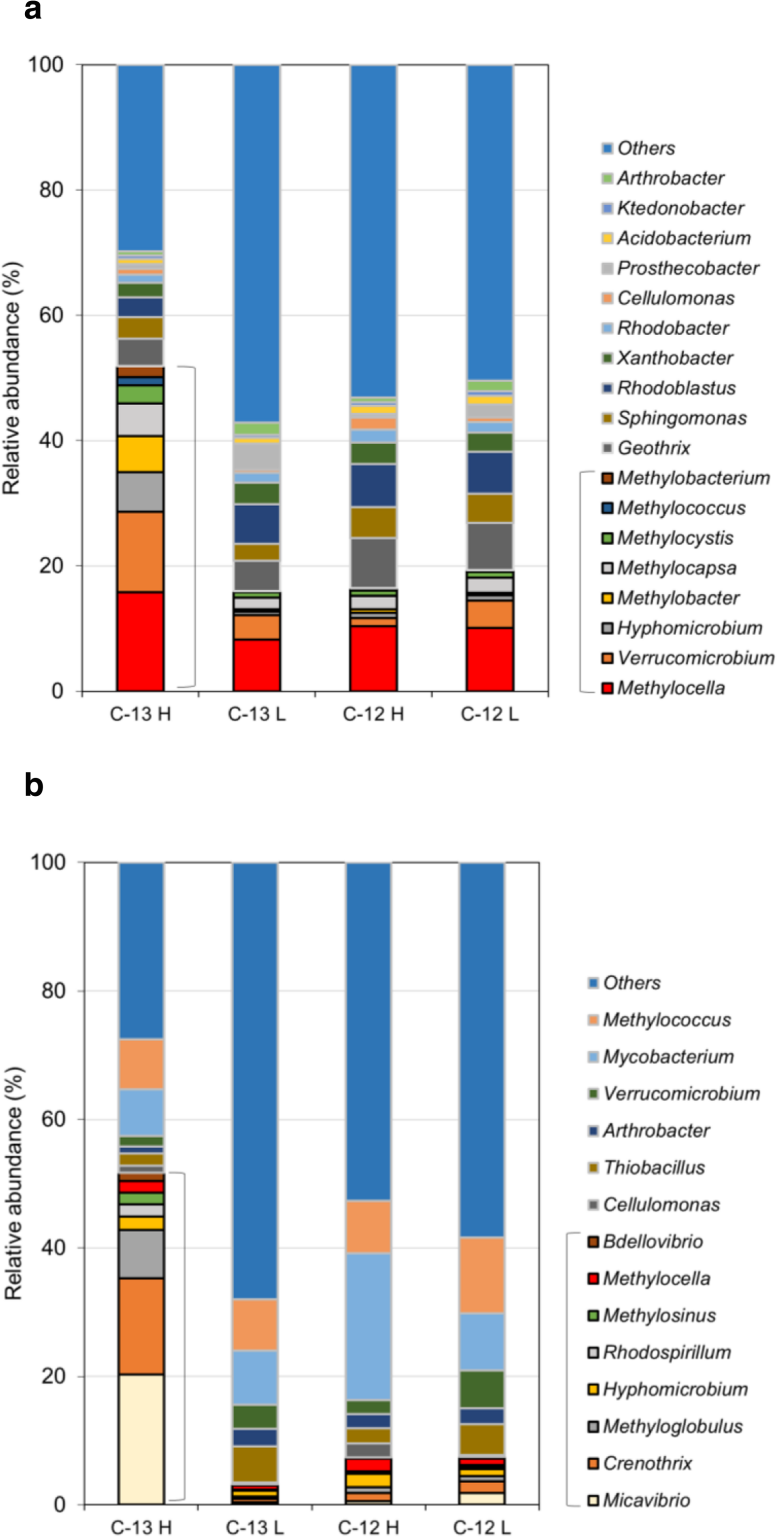
Community profile of the enriched heavy (C-13 H) and light (C-13 L) DNA fractions of ^13^C-methane incubations from DNA-SIP experiment with the Pipe Creek samples (**a**) and Andreiasu Everlasting Fire samples (**b**), analysed by 16S rRNA gene amplicon sequencing. Sequencing community profiles of heavy (C-12 H) and light (C-12 L) fractions of control incubations with ^12^C-methane are also presented. Taxa represented by black borders and in parenthesis are identified as “^13^C-labelled” in that experiment. Taxa present at a relative abundance lower than 1% in any replicate of C-13 H fraction are included in “Others”. Data presented here are the mean of biological duplicates

The 16S rRNA gene sequences retrieved by PCR from heavy and light DNA fractions of ^13^C-methane- and ^12^C-methane-incubated samples were resolved into operational taxonomic units (OTUs) at the genus level. Eighteen OTUs (Pipe Creek) and 14 OTUs (Andreiasu Everlasting Fire) were found at a relative abundance of greater than 1% in heavy DNA fractions retrieved from ^13^C-methane-incubated samples (Fig. 3). Taxa were identified as being ^13^C-labelled based on their relative abundance in heavy and light fractions of incubations with ^13^C-methane, as compared with ^12^C-methane controls (as described in the “Materials and methods” section). The relative abundance of labelled taxa constituted 51.84% and 51.65% of the total in Pipe Creek and Andreiasu Everlasting Fire, respectively. Sequences affiliated with the genera *Methylocella* (30.3%), *Verrucomicrobium* (24.8%) *Methylobacter* (11.1%), *Methylocapsa* (10.0%) and *Methylocystis* (5.5%) were detected in ^13^C-labelled DNA obtained from DNA-SIP experiments with samples from Pipe Creek (Fig. 3), identifying *Methylocella* as the most abundant active methanotroph at this site. *Methylocella* were also ^13^C-labelled in Andreiasu Everlasting Fire DNA-SIP samples and constituted 3.5% of ^13^C-labelled taxa along with the methanotrophs *Crenothrix* (28.9%), *Methyloglobulus* (14.6%) and *Methylosinus* (3.6%) (Fig. 3). *Hyphomicrobium* and *Methylobacterium* were the only non-methanotrophs identified as ^13^C-labelled in DNA-SIP experiments with Pipe Creek samples, while in DNA-SIP experiments with Andreiasu Everlasting Fire samples, non-methanotrophic bacteria labelled with ^13^C were *Hyphomicrobium*, *Rhodospirillum*, *Micavibrio* and *Bdellovibrio* (Fig. 3). *Hyphomicrobium* and *Methylobacterium* are methylotrophic bacteria and may have been feeding on methanol released during methane utilisation by methanotrophs [86]. *Micavibrio* and *Bdellovibrio* are bacterial predators that might also have been cross-feeding on active methanotrophic bacteria. The presence and labelling of methylotrophs suggest that methanotrophs are supporting a community of non-methane oxidisers in these environments. Our data show that *Methylocella* was the only active methanotrophic genus common to both environments. Previously, *Methylocella* were detected in environmental samples originating from acidic forest soils and acidic peatlands as reported in a few cultivation-independent [87–90] and cultivation-dependent studies [54, 91–96]. Studies focussing on functionally active methanotrophs (for example [97–100]) also detected *Methylocella* in acidic environments leading to the belief that *Methylocella* were mainly confined to acidic environments, although, interestingly, some studies reported that *Methylocella* were also abundant in alkaline environments [58, 59, 101]. Our observation that *Methylocella* are active methanotrophs in environmental samples from geological natural gas-emitting sites of acidic and basic pH suggest that its metabolic flexibility gives *Methylocella* a competitive advantage over other methanotrophs to utilise methane and other short-chain alkanes in such environments.

### Targeted isolation of facultative methanotrophs

As indicated above, culture-independent DNA-SIP experiments showed that *Methylocella*-like facultative methanotrophs were one of the most abundant and active methanotrophs at the natural gas seeps. To complement these results, we isolated facultative methanotrophs capable of utilising methane, ethane and propane as their only source of carbon and energy. Enrichments of environmental samples from the seep sites were incubated under a mixture of methane, ethane and propane (in a proportion comparable to the gas released from Pipe Creek). Serial dilutions of enrichment cultures were plated and incubated under the gas mixture, and colonies were screened for parallel growth on each gas separately. We isolated two bacterial strains growing under these gases, both as a mixture and individually (Table 2, Additional file 1: Table S1). *Methylocella* sp. PC1 and PC4 from Pipe Creek exhibited faster growth rates on methane and propane (up to 0.04 h^−1^, Table 2) compared to those previously reported for *Methylocella* (0.01–0.02 h^−1^ on methane and 0.005–0.015 h^−1^ on propane) [49, 102, 103]. As with *Methylocella silvestris* BL2 [49], they grew under methane and propane simultaneously and could also utilise non-gaseous multicarbon compounds, including acetate, pyruvate and succinate (Additional file 1: Table S1). The isolates grew faster on ethane compared to their growth on methane or propane (Table 2, Additional file 1: Figure S3), as the only source of C and energy. Since these isolates were from an environment rich in ethane [18, 59], they may have been adapted to ethane utilisation and, hence, can utilise the major components of natural gas and serve as a natural biofilter for the various geological gases before they are emitted from terrestrial natural gas seep sites to the atmosphere.

**Table 2:** Growth rates^1^ of new *Methylocella* isolates with methane and other short-chain alkanes

Isolate	Methane (20 %, v/v)	Ethane (10 %, v/v)	Propane (10 %, v/v)
*Methylocella* sp. PC1	4.1 × 10^−2^ (± 5.0 × 10^−5^)	5.7 × 10^−2^ (± 7.0 × 10^−4^)	3.4 × 10^−2^ (± 8.0 × 10^−4^)
*Methylocella* sp. PC4	4.1 × 10^−2^ (± 5.0 × 10^−5^)	5.3 × 10^−2^ (± 5.0 × 10^−4^)	3.4 × 10^−2^ (± 1.5 × 10^−4^)

^**1**^Cultures were grown in 20 ml volume in 120 ml sealed serum vials. Growth was measured in terms of optical density increase (measured at 540 nm). Growth rates (per hour) are calculated using at least four growth points of logarithmic growth phase (Additional file 1: Figure S3). Values presented are biological duplicates (± standard error of means).

### Functional genomics and comparative genomics of *Methylocella* isolates

**Fig. 4:**
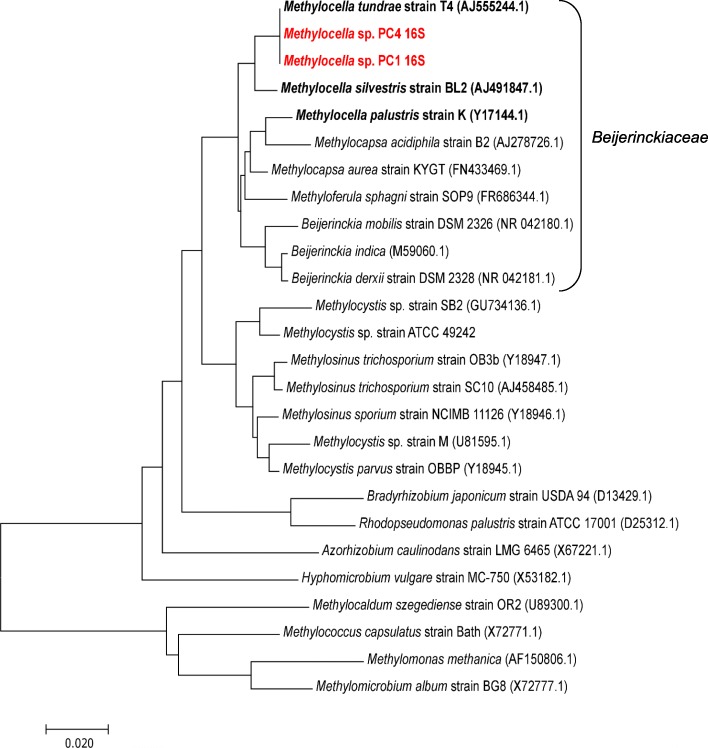
Phylogenetic analysis based on 16S rRNA sequences from new *Methylocella* isolates (bold red) along with other known *Methylocella* (bold black) strains. Accession number for the nucleotide sequences are given in brackets. Sequences were aligned using Mega 7.0 and the optimal tree (drawn to scale, with branch lengths measured in the number of substitutions per site) with the sum of branch length = 0.65 is shown where the evolutionary history was inferred using the neighbour-joining method (1067 positions in the final dataset)

Analysis based on the 16S rRNA gene revealed that strains *Methylocella* sp. PC1 and PC4 were most closely affiliated with *Methylocella tundrae* (Fig. 4). Previously, complete genomes of only three strains of *Methylocella* have been reported, belonging to *Methylocella silvestris* and *Methylocella tundrae* species [55, 104, 105]. Genome sequencing and in silico DNA-DNA hybridization analyses suggest that these novel *Methylocella* sp. PC1 and PC4 strains represent subspecies of *Methylocella tundrae* distinct from *Methylocella tundrae* T4 strain (Additional file 1: Table S2).

PCR analyses as well as genome sequence analysis of the isolates showed that the *mmoX* gene (encoding MmoX, the alpha subunit of sMMO) is present while the *pmoA* gene (encoding the alpha subunit of pMMO) is absent. This is in agreement with previous reports of *Methylocella* [55, 104]. Apart from *Methylocella*, the obligate methanotrophs *Methyloceanibacter* [106] and *Methyloferula stellata* [107], also lack pMMO and rely on sMMO for methane oxidation.

**Fig. 5:**
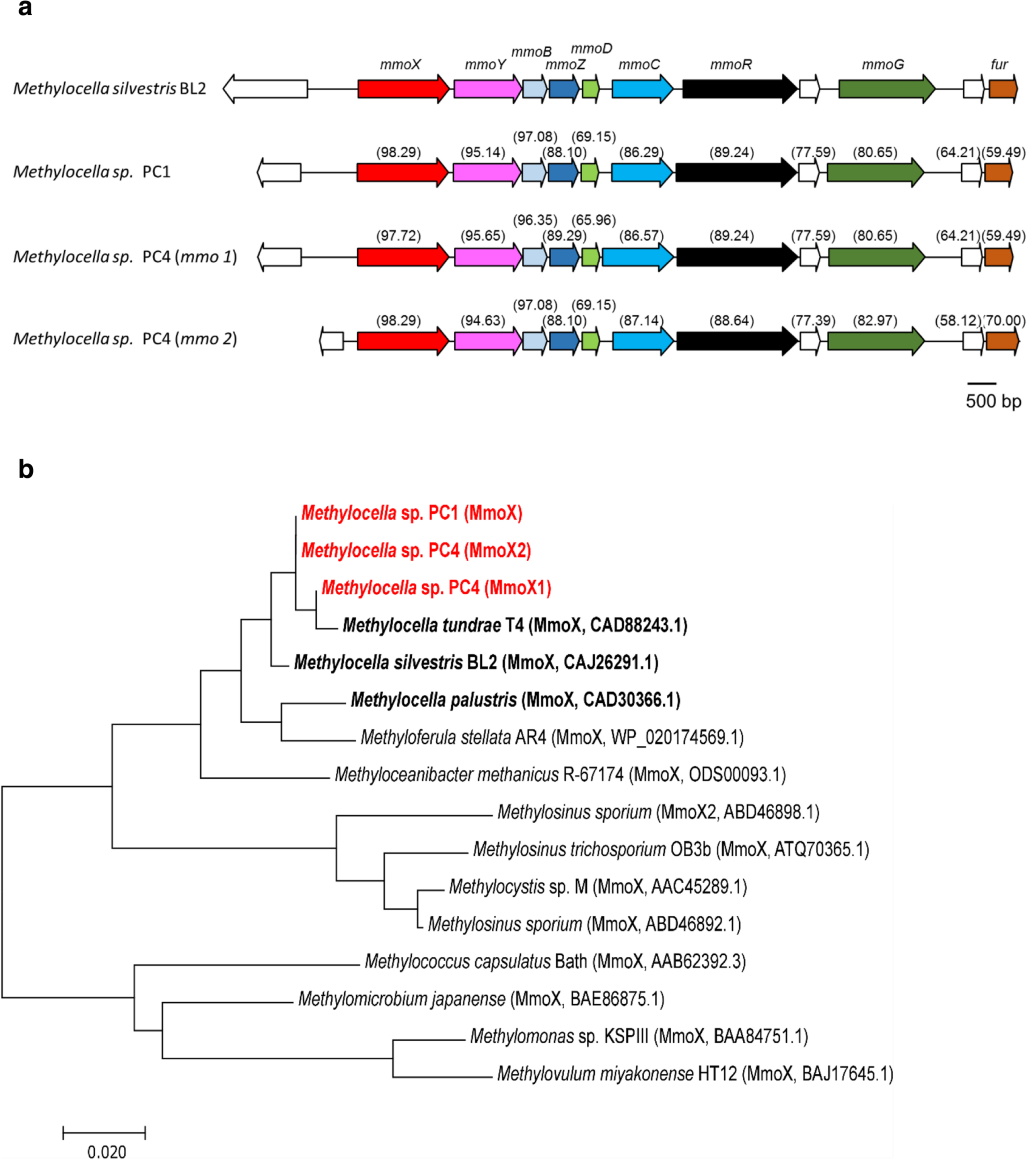
**a** The soluble methane monooxygenase genes in *Methylocella* sp. PC1 and PC4 isolates compared with their homologues in *Methylocella silvestris* BL2. Names of the genes are given above, and amino acid identities to their homologous proteins in *Methylocella silvestris* BL2 are shown in brackets. **b** Phylogenetic analysis of MmoX (alpha subunit of soluble methane monooxygenase) from new *Methylocella* isolates (bold red) along with MmoX from other known *Methylocella* (bold black) strains based on the derived amino acid sequences. Protein names and accession numbers for the sequences are given in brackets. Amino acid sequences were aligned using Mega 7.0 and the optimal tree (drawn to scale, with branch lengths measured in the number of substitutions per site) with the sum of branch length = 0.53 is shown where the evolutionary history was inferred using the neighbour-joining method (392 positions in the final dataset). The percentage of replicate trees in which the associated taxa clustered together in the bootstrap test (1000 replicates) ranged from 59 to 100

Detailed analyses of the genomes revealed the presence of the genes of the soluble methane monooxygenase (*mmo* operon genes), in both isolates (Fig. 5). In contrast to *Methylocella silvestris* BL2 that has only one copy of the *mmo* operon, two complete *mmo* operons are present in *Methylocella* sp. PC4 (Fig. 5). To our knowledge, having multiple copies of *mmo* operons is unique to this novel *Methylocella* sp. PC4 isolate compared to other extant *Methylocella* strains. The *mmo* operons in *Methylocella* sp. PC1 and PC4 are highly conserved and of high similarity to those present in *Methylocella silvestris* BL2 (Fig. 5). The structural genes for sMMO (*mmoXYBZDC*) are adjacent to regulatory genes encoding a σ^54^ transcriptional regulator (*mmoR*) and a putative GroEL-like chaperone (*mmoG*), respectively.

**Fig. 6:**
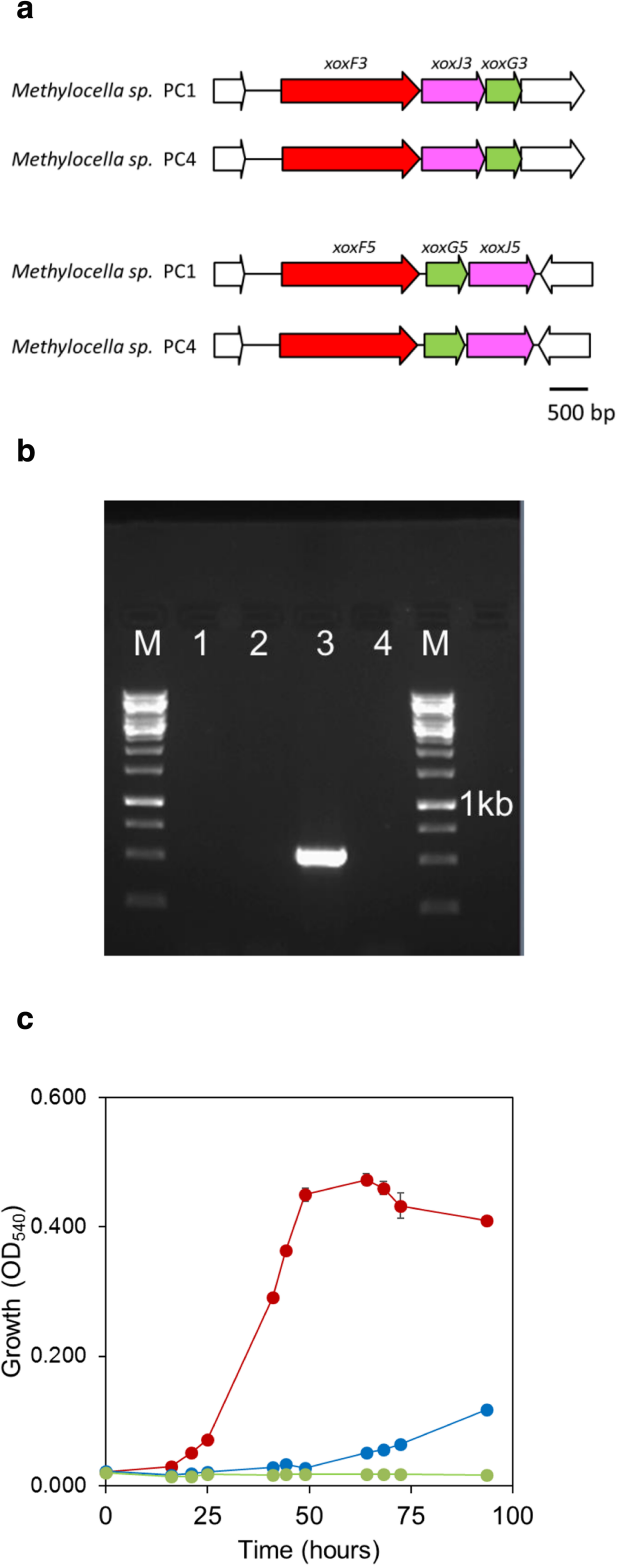
**a** Gene clusters encoding for the two types of lanthanide-dependent methanol dehydrogenases XoxF3 and XoxF5 in *Methylocella* sp. PC1 and PC4. Names of the genes are given above. Another copy of *xoxF5* is also present in the genomes of *Methylocella* PC1 and PC4, but only as a singleton gene (see Additional file 1: Figure S4). **b** PCR amplification showing the absence of *mxaF* in *Methylocella* sp. PC1 (lane 1) and *Methylocella* sp. PC4 (lane 2) along with positive control *Methylocella silvestris* BL2 (lane 3) and no template control (lane 4). Lane M represents 1 kb DNA ladder. **c** Growth of *Methylocella* sp. PC4 with (red) and without (blue) lanthanum. Cultures were grown in DNMS medium with methanol (20 mM) as the only source of carbon and energy. Each point shows the average of duplicate cultures with error bars (invisible if smaller than symbol size) showing the standard errors. A control without any carbon substrate showing no growth of cells (green) was also performed

Methanol dehydrogenase, the second essential enzyme for methane metabolism, catalyses the conversion of methanol to formaldehyde during C-1 metabolism. Surprisingly, *Methylocella* sp. PC1 and PC4 lacked the *mxa* gene operon encoding the classical calcium-containing methanol dehydrogenase. Instead, multiple copies of the *xox* gene clusters encoding for a lanthanide-containing methanol dehydrogenase XoxF and associated proteins (XoxJ and XoxG) were present (Fig. 6a). Two complete *xox* gene operons (*xoxFJG*) phylogenetically related to XoxF5 and XoxF3 clades [108] are present (Additional file 1: Figure S4). Absence of calcium-containing methanol dehydrogenase in *Methylocella* sp. PC1 and PC4 was confirmed by failure to PCR-amplify *mxaF*, encoding the alpha subunit (Fig. 6b). *Methylocella* sp. PC1 and PC4 showed very poor growth with methanol as the only source of C and energy when grown without adding lanthanides in the growth medium (Fig. 6c), confirming lanthanide-dependence under these conditions. All *Methylocella* strains and other methanotrophs described to date contain the classical calcium-containing methanol dehydrogenase, usually in addition to the lanthanide-dependent methanol dehydrogenase(s) [33, 34, 47], with the exception of the *Verrucomicrobium* methanotroph *Methylacidiphilum fumariolicum* SolV [36] and two gammaproteobacterial methanotrophs [109]. The recently discovered role of lanthanides in the activity and regulation of methanol dehydrogenase had prompted us to use lanthanum as a regular nutrient in the medium for enrichment and isolation of methanotrophs, which facilitated the isolation of novel *Methylocella* sp. PC1 and PC4 isolates containing only lanthanide-dependent methanol dehydrogenase. To our knowledge, these are the first alphaproteobacterial methanotrophs which do not contain a calcium-containing methanol dehydrogenase.

In addition to *mmo* and *xox* gene operons, other genes required for the central metabolism of C-1 substrate were also analysed (Additional file 1: Table S3). A complete set of genes encoding enzymes required to convert formaldehyde into formate via tetrahydromethanopterin (H_4_MPT) pathway was found (Additional file 1: Table S3), and the genes (*mtdA* and *fchA* encoding methylene-tetrahydrofolate dehydrogenase and methenyl-tetrahydrofolate cyclohydrolase, respectively) required to convert formaldehyde into formate via the tetrahydrofolate (H_4_F) pathway were not found, but instead *folD*, encoding bifunctional 5,10-methylene-H_4_F dehydrogenase/methenyl-H_4_F cyclohydrolase, was present. For oxidation to CO_2_, genes encoding molybdenum-containing formate dehydrogenase (FDH2) [110] were found (Additional file 1: Table S3). To assimilate formaldehyde into biomass, the genes encoding enzymes of the serine cycle were present in both isolates (Additional file 1: Table S3). Our comparative genomic analyses show that the pathway for the assimilation and oxidation of formaldehyde from C-1 substrates to produce biomass and energy is similar in these isolates to that of *Methylocella silvestris* BL2 (Additional file 1: Table S3).

**Fig. 7:**
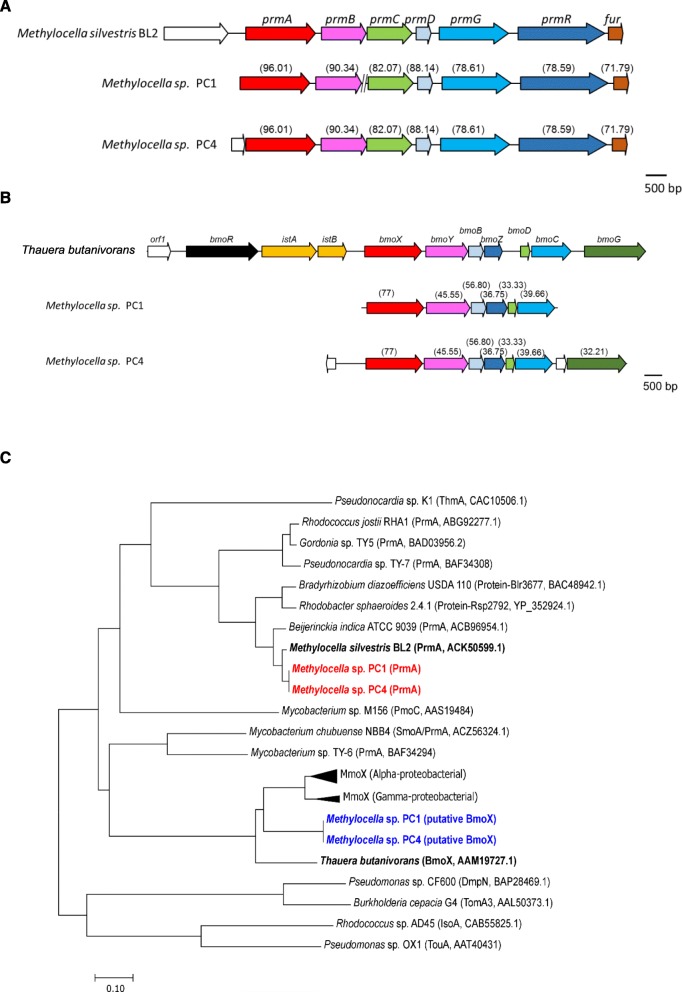
**a** Gene operon encoding the propane monooxygenase in *Methylocella* sp. PC1 and PC4 compared with their homologues in *Methylocella silvestris* BL2. Names of the genes are given above and amino acid identities to their homologous proteins in *Methylocella silvestris* BL2 are shown in brackets. **b** Gene operon putatively encoding butane monooxygenase in *Methylocella* sp. PC1 and PC4 compared with their homologues in *Thauera butanivorans*. **c** Phylogenetic analysis of the alpha subunit of propane monooxygenase (PrmA) and putative butane monooxygenase (BmoX) from *Methylocella* PC1 and PC4 isolates (red and blue, respectively). PrmA and BmoX from known closely related strains (bold black) and other soluble diiron monooxygenases (ThmA, alpha subunit of tetrahydrofuran monooxygenase; Blr3677 and Rsp2792, putative monooxygenases; PmoC, alpha subunit of propene monooxygenase; MmoX, alpha subunit of methane monooxygenase; DmpN, alpha subunit of phenol hydroxylase; TomA3, alpha subunit of toluene ortho-monooxygenase; IsoA, alpha subunit of isoprene monooxygenase; and TouA, toluene o-xylene monooxygenase component) from different bacteria are also presented. Compressed MmoX sequences are same as the known methane monooxygenases presented in Fig. 5b. Protein names and accession number for the sequences are given in brackets. Amino acid sequences were aligned using Mega 7.0 and the optimal tree (drawn to scale, with branch lengths measured in the number of substitutions per site) with the sum of branch length = 5.7 is shown where the evolutionary history was inferred using the neighbour-joining method (316 positions in the final dataset). The percentage of replicate trees in which the associated taxa clustered together in the bootstrap test (1000 replicates) ranged from 60 to 100

The genomes of *Methylocella* sp. PC1 and PC4 contained genes (*prmABCDGR*) encoding PrMO, a SDIMO enzyme responsible for the growth of bacteria on short-chain alkanes (Fig. 7a). Genes encoding PrMO in *Methylocella* sp. PC1 and PC4 share identities (> 78% based on AA sequences) with those found in *Methylocella silvestris* BL2. *Methylocella* silvestris BL2 and *Methylocella silvestris* TVC are the only strains described previously, capable of growing on methane and propane and containing both sMMO and PrMO [55, 104]. Interestingly, and in contrast to other *Methylocella* strains, isolates *Methylocella* sp. PC1 and PC4 contain more divergent copies of gene clusters putatively encoding another SDIMO in addition to PrMO and sMMO. BLAST and phylogenetic analyses reveal that these gene clusters (here named as *bmoXYBZDCG* in *Methylocella* sp. PC1 and PC4) are more similar to the *bmo* genes encoding butane monooxygenase found in *Thauera butanivorans* (Fig. 7b, c) [111, 112]. *Methylocella* sp. PC1 and PC4 did not grow with butane as the only source of carbon and energy. The reason could be that this *bmo*-like gene cluster in both *Methylocella* sp. PC1 and PC4 lacks *bmoR and istAB* genes (Fig. 6b). BmoR is putatively involved in the regulation of butane monooxygenase and was required for good growth of *Thauera butanivorans* on butane [112]. However, the presence of multiple SDIMOs in the genomes of *Methylocella* sp. PC1 and PC4, in particular, sMMO and PrMO enabled them to grow on methane as well as on ethane and propane, the major components of fugitive nature gas. This report is to our knowledge the first to describe and to isolate novel strains of facultative methanotrophs from natural gas seep sites. Considering the extent of gas seeps spread over Earth’s terrestrial regions, this finding has profound implications for the biological consumption of natural gas and carbon cycling in these environments, which have been ignored in the past.

## Conclusion

Here, we provide evidence, using culture-dependent and culture-independent methods, that *Methylocella* are abundant and active at terrestrial natural gas seeps. *Methylocella* were the only active methanotroph found in both of the contrasting natural gas seep sites tested, suggesting that they play a significant role in biogeochemical cycling of these gaseous alkanes and may serve as a natural biofilter for gaseous hydrocarbons of geological sources before they are emitted to the atmosphere.

We also isolated novel *Methylocella* isolates with considerable differences to extant strains, illustrating that natural gas seeps may be a rich source of new methanotrophs. Using lanthanum as a nutrient in medium for the enrichment of cultures from the natural gas seep sites we isolated *Methylocella* sp. PC1 and PC4, which contain only XoxF methanol dehydrogenase. Our comparative genomic and growth data suggest that the ability of *Methylocella* strains to utilise methane as well as short-chain alkanes, integral components of natural gas, is not restricted to one species.

The bacteria obtained in this study provide novel experimental models for investigating the complexity and function of the facultative methanotrophic community active at terrestrial natural gas seeps. This would also be of significance for the design of environmental biotechnological strategies for controlling natural gas emissions along with industrial applications for converting these gases to value-added products, as natural gas emissions are increasing globally due to unconventional oil and gas extraction.

## Materials and methods

### Chemicals and reagents

All chemicals and reagents (purity, > 99%) were obtained from Sigma-Aldrich unless otherwise stated. Buffers, culture media and solutions were prepared in ultra-pure water, and sterilisation was done by autoclaving (15 min, 121 °C, 1 bar) or by filtration (0.2 μm).

### Bacterial strains and growth conditions

Modified diluted nitrate mineral salt (DNMS) medium [49] supplemented with 5 μM lanthanum (LaCl_3_) was used as a growth medium in 120 ml serum vials (with 20 ml culture volume), with substrate gas as the only source of C and energy. Methane (20%), ethane (10%) and propane (10%), individually or in a mixture at varying concentrations, were injected (percentage v/v in headspace) in sealed serum vials as C substrates. The growth of liquid cultures was monitored by measuring the optical density at 540 nm. Concentrations of substrate gases in the cultures were quantified using a gas chromatograph (GC) using an Agilent 7820A GC equipped with a Porapak Q column (Supelco) coupled to a flame ionisation detector (FID) to measure methane, ethane and propane concentrations as previously described [49]. Comparison of growth of *Methylocella* sp. PC4 with and without lanthanum (Fig. 6) was performed in 50 ml polypropylene falcon tubes (with 15 ml culture volume) to avoid any contamination of lanthanides from glassware. As many methanotrophs do not store well frozen [113], cultures were maintained on plates or in liquid.

### Sample collection

Two different natural gas seep sites, (Andreiasu Everlasting Fire, Romania (Additional file 1: Figure S1B), with a slightly basic pH (8.2), and Pipe Creek, New York, USA (Additional file 1: Figure S1B), with slightly acidic pH (6.0)) with varying characteristics of physical nature, pH and the proportion of methane, ethane and propane in the gas released, were sampled as described previously [59]). Two to five sub-samples, taken from each site in sterile 50 ml plastic tubes, were pooled together in the lab before further experiments were carried out.

### DNA-SIP incubation, DNA extraction and fractionation

For DNA-SIP experiments, approximately 2 g of soil/sediment suspensions (1:3 environmental samples and water ratio) in sterile ultra-pure molecular biology grade water without any nutrient supplements were incubated in 120 ml sealed serum vials. Substrate gas (^12^C-methane or ^13^C-methane) was injected into the headspace of each serum vial at 1% concentration (v/v) and consumption was followed over time by GC. Time point 1 samples from DNA-SIP incubations were harvested after they had consumed approximately 1% (v/v) added gas, and for time point 2 samples, ^12^C-methane or ^13^C-methane was replenished by injecting an extra 1% (v/v) of gas and then harvested after approximately 2% (v/v) of total gas had been consumed. Samples were harvested in order to obtain time points based on 0, 1% (v/v) and 2% (v/v) ^12^C-methane or ^13^C-methane gas consumed, corresponding to 0, 100 and 200 μmol C consumed per gram of fresh sample, respectively. All incubations were carried out in duplicate for each substrate and for each time point. Samples were harvested by centrifugation at 10,000 × *g* for 15 min in 50 ml falcon tubes. Supernatants were discarded and the soil/sediment pellets were stored at − 20 °C and used later for DNA extraction. DNA was extracted from unenriched environmental samples and SIP-incubated samples using the FAST DNA spin kit for soil (MP Biomedicals), following the manufacturer’s instructions. Quality and quantity of DNA was checked by Qubit (Invitrogen) and NanoDrop (Thermo Fisher Scientific).

Labelled and unlabelled DNA from SIP-incubated samples were separated by density gradient ultracentrifugation and fractionation (12 fractions per sample) as described previously [114]. The quantity of DNA retrieved from each fraction was plotted against the corresponding refractive index, quantified using a refractometer (Reichert AR200, Reichert Analytical Instruments, Buffalo, NY, USA) (Additional file 1: Figure S2). Based on the data shown in Additional file 1: Figure S2, three to four fractions of each sample containing labelled DNA (refractive index range 1.4032–1.4045) were mixed and designated as the “heavy” DNA fraction, while two to three fractions of each sample containing unlabelled DNA (refractive index range 1.4015–1.4025) were mixed and designated as the “light” DNA fraction.

### Illumina Mi-Seq sequencing of PCR amplicons

PCR amplicons of 16S rRNA gene obtained from the unfractionated unenriched environmental DNA samples and the heavy and light DNA fractions from ^12^C-methane- or ^13^C-methane-incubated samples, were sequenced using the Illumina Mi-Seq sequencing platform of MR DNA (Shallowater, TX, USA). Universal primers 341F and 785R [115] targeting the V3 and V4 regions were used to PCR amplify 16S rRNA gene fragment in a reaction volume of 25 μl containing 12.5 μl 2x PCRBIO Ultra Polymerase (PCR BIO), 1 μl of each of forward and reverse primers (10 μM) and 2 μl of template DNA. The cycling conditions were 95 °C for 3 min, followed by 30 cycles of 94 °C for 20 sec, 55 °C for 20 sec, 72 °C for 30 sec, with a final extension at 72 °C for 5 min. PCR products from duplicate reactions for each fraction were pooled before purifying using a NucleoSpin gel and PCR Clean-up kit (Macherey-Nagel). The quality and quantity of purified PCR products was assessed by gel electrophoresis and a NanoDrop and then concentrations of all PCR products were adjusted to 15–20 ng/μl. DNA libraries following the Illumina TruSeq DNA library protocol from purified PCR products were prepared and sequenced. Sequence data from 16S rRNA amplicons was processed using the MR DNA proprietary analysis pipeline (www.mrdnalab.com) as described previously [59]. Briefly, barcode and primer sequences were removed and then short sequences < 200 bp, sequences with ambiguous base calls and sequences with homopolymer runs exceeding 6 bp were removed. After denoising of the sequences, 16S rRNA gene OTUs were defined with clustering at 3% divergence (97% identity) followed by removal of singleton sequences and chimaeras [116–121]. BLASTn against a curated database derived from GreenGenes, RDPII and NCBI (www.ncbi.nlm.nih.gov, http://rdp.cme.msu.edu) was used for the final taxonomic classification of OTUs into each taxonomic level. Taxa fulfilling the following criteria were identified as labelled: (1) the relative abundance in the heavy DNA fraction of the ^13^C-methane-incubated sample was higher than 1.0%, (2) the abundance in the heavy DNA fraction of the ^13^C-methane-incubated sample was higher than the abundance in the light DNA fraction of the ^13^C-methane and (3) the difference in the abundance in the compared heavy and light DNA fractions of the ^13^C-methane-incubated sample was higher than that of the ^12^C-methane-incubated sample.

### Enrichment cultures, isolations and genome sequencing of new *Methylocella* isolates

Fresh samples (1 g) from the Pipe Creek natural gas seep site were incubated in 10 ml DNMS medium supplemented with 5 μM lanthanum in 120 ml sealed serum vials. A mixture of gases (20%, v/v) in the headspace was injected as the only supplemental source of C and energy comprising of methane, (70%, v/v) ethane (10%, v/v) and propane (20%, v/v), and the vials were incubated in a shaker (150 rpm) at 25 °C for 3 weeks in the dark. Then, serial dilutions (1/10 times) of the enrichment cultures were plated onto DNMS agar plates (supplemented with 5 μM lanthanum), and incubated again under a mixture of gases (10%, v/v) injected into the headspace containing methane (70%, v/v) ethane (10%, v/v) and propane (20%, v/v) in a sealed jar. After 2 weeks of incubation, isolated colonies appearing on the plates were resuspended in 20 μl sterile DNMS medium individually. Each colony suspension was replica-plated onto three plates of DNMS medium (supplemented with 5 μM lanthanum) and incubated under methane, ethane or propane individually in the headspace (10%, v/v) as the only source of carbon and energy. Colonies growing under all three gases were considered as facultative methanotrophs. These facultative methanotrophs were purified by serial dilution of cultures and transfer of single colonies several times on agar plates. Purity of cultures was confirmed by microscopy of cultures, multiple cloning and sequencing of 16S rRNA and *mmoX* genes and subsequent genome sequencing. The genome sequences were checked for completeness and contamination using CheckM [122]. Upon mapping of raw reads to the assembly using Bowtie2 (v. 2.3.4.1) [123], ~ 99% of all reads aligned to the genome, thus confirming purity of these new *Methylocella* strains (Additional file 1: Table S5). Genomic DNA from *Methylocella* strains was extracted using the Wizard Genomic DNA Purification Kit (Promega) according to the manufacturer’s instructions and used for PCR analyses to generate 16S rRNA, *mmoX*, *mxaF*, *pmoA* and *prmA* amplicons using specific primers (Additional file 1: Table S4). Genome sequencing of *Methylocella* PC1 and PC4 was performed at MicrobesNG (Birmingham, UK) using Illumina HiSeq technology and assembled using SPAdes 3.7 into contigs (Additional file 1: Table S5). Genome sequence annotation, exploration and comparative genomics of various *Methylocella* strains were performed using MicroScope, an online platform by GenoScope (France) providing a collection of bioinformatic tools [124].

## Additional file

Additional file 1: **Table S1**. Growth of *Methylocella* sp. PC1 and PC4 under various substrates. **Table S2**. In-silico DNA-DNA hybridization (DDH) of *Methylocella* sp. PC1 and PC4 genomes compared with other *Methylocella* strains. **Table S3**. Genes identified putatively involved in the central metabolism of C-1 substrates in *Methylocella* sp. PC1 and PC4. **Table S4**. Primers used for PCR amplification in this study. **Table S5**. Characteristics of sequenced genomes of new *Methylocella* isolates. **Figure S1**. Relative abundance (%) of dominant bacterial phyla as revealed by 16S rRNA gene sequencing of DNA from native environmental samples. **Figure S2.** DNA retrieved as a function of refractive index of each fraction recovered after ultracentrifugation. **Figure S3.** Growth curves of *Methylocella* sp. PC1 and PC4 under various gaseous substrates. **Figure S4.** Phylogenetic analysis of methanol dehydrogenases from *Methylocella* PC1 and PC4 isolates. (PDF 798 kb)

## Data Availability

Amplicon sequence data generated in this study were deposited to sequence read archives (SRA) under project number PRJNA525613 and the genome sequence assemblies have deposited to European Nucleotide Archives under project numbers PRJEB31473 and PRJEB31475. Strains are available from JCM/MFUH on request.
