# Supplementary material for: Novel facultative Methylocella strains are active methane consumers at terrestrial natural gas seeps
Source: Microbiome. 2019 Oct 4;7:134. doi: 10.1186/s40168-019-0741-3 (PMC6778391; doi:10.1186/s40168-019-0741-3)
Supplement: Supplementary file 1 — Table S1. Growth of Methylocella sp. PC1 and PC4 under various substrates. Table S2. In-silico DNA-DNA hybridization (DDH) of Methylocella sp. PC1 and PC4 genomes compared with other Methylocella strains. Table S3. Genes identified putatively involved in the central metabolism of C-1 substrates in Methylocella sp. PC1 and PC4. Table S4. Primers used for PCR amplification in this study. Table S5. Characteristics of sequenced genomes of new Methylocella isolates. Figure S1. Relative abundance (%) of dominant bacterial phyla as revealed by 16S rRNA gene sequencing of DNA from native environmental samples. Figure S2. DNA retrieved as a function of refractive index of each fraction recovered after ultracentrifugation. Figure S3. Growth curves of Methylocella sp. PC1 and PC4 under various gaseous substrates. Figure S4. Phylogenetic analysis of methanol dehydrogenases from Methylocella PC1 and PC4 isolates. (PDF 798 kb) [file 40168_2019_741_MOESM1_ESM.pdf]

(Additional file 1)

Novel facultative *Methylocella* strains are active methane consumers at terrestrial natural gas seeps

Muhammad Farhan Ul Haque[1,*], Andrew T Crombie[2,*], J Colin Murrell[1]

Author's affiliations

[1] School of Environmental Sciences, University of East Anglia, Norwich, UK

[2] School of Biological Sciences, University of East Anglia, Norwich, UK

* Correspondence:

M.Farhan@uea.ac.uk

School of Environmental Sciences, University of East Anglia, Norwich Research Park, NR4 7TJ, Norwich, UK

A.Crombie@uea.ac.uk

School of Biological Sciences, University of East Anglia, Norwich Research Park, NR4 7TJ, Norwich, UK

Table S1. Growth of *Methylocella* sp. PC1 and PC4 under various substrates

Substrate (concentration)	Substrate type	*Methylocella* sp. PC1*	*Methylocella* sp. PC4*
Methane (10% to 20%)	C-1	++	++
Methanol (0.05% to 1%)	C-1	++	++
Ethane (2% to 10%)	C-2	++	++
Ethene (5%)	C-2	-	-
Ethanol (0.05%)	C-2	++	++
Propane (2% to 10%)	C-3	++	++
Propene (5%)	C-3	-	-
1-Propanol (0.05%)	C-3	-	-
2-Propanol (0.05%)	C-3	++	++
Methane + Ethane + Propane (various)	C1+C2+C3	++	++
Butane (10%)	C4	-	-
Butanol (0.05%)	C4	-	-
Glucose (5 mM)	multi-carbon	++	++
Fructose (5 mM)	multi-carbon	++	++
Acetate (5 mM)	multi-carbon	+	++
Succinate (5 mM)	multi-carbon	++	++
Pyruvate (5 mM)	multi-carbon	++	++
Formate (5 mM)	multi-carbon	+	+
Citrate (5 mM)	multi-carbon	-	-
Glycerol (5 mM)	multi-carbon	++	++

*Growth is indicated as: ++ growth to $OD_{540} > 0.20$; + growth to $OD_{540} \geq 0.08$–0.20; − $OD_{540} < 0.08$. (Concentrations in % are given as v/v.). All cultures were cultivated in DNMS medium supplemented with lanthanum (5 µM).

Table S2. ***In-silico*** **DNA-DNA hybridization (DDH) of** ***Methylocella*** **sp. PC1 and PC4 genomes compared with other** ***Methylocella*** **strains using Genome-to-Genome Distance Calculator 2.1[1].**

	% DDH[2,3]		
**Strains**	*Methylocella silvestris* BL2	*Methylocella* sp. TVC	*Methylocella tundrae* T4
Methylocella sp. PC1	22.90%	23.00%	79.00%
Methylocella sp. PC4	21.30%	21.10%	78.40%

[1] Genome-to-Genome Distance Calculator 2.1 (http://ggdc.dsmz.de/distcalc2.php) [1].

[2] DDH estimated % values are analogous to DDH using a generalized linear model inferred from an empirical reference dataset comprising real DDH values and genome sequences.

[3]To delineate microbial subspecies, a cut-off of 80% DDH was used [2]. If the *in-silico* DDH value between two strains is 70% to 80%, then they are considered as subspecies within the same species.

Table S3. Genes identified putatively involved in the central metabolism of C-1 substrates in *Methylocella* sp. PC1 and PC4.

Gene name	Gene Product	*Methylocella* sp. PC4	*Methylocella* sp. PC1	*Methylocella silvestris* BL2
**Genes required to carry out serine pathway for the assimilation of C from C-1 substrates**				
ftfL	Formate-tetrahydrofolate ligase (formyltetrahydrofolate synthetase)	MPC4_150003	+	+
hprA	Hydroxypyruvate reductase, NAD(P)H-dependent	MPC4_150004	+	+
sgaA	Serine-glyoxylate aminotransferase	MPC4_150005	+	+
gck	Glycerate kinase	MPC4_150006	+	+
mtkA	Malate thiokinase, large subunit	MPC4_150007	+	+
mtkB	Malate thiokinase, small subunit B (Malate-CoA ligase)	MPC4_150008	+	+
ppc	Phosphoenolpyruvate carboxylase	MPC4_150010	+	+
mclA	L-malyl-CoA/beta-methylmalyl-CoA lyase	MPC4_150011	+	+
glyA	Serine hydroxymethyltransferase	MPC4_50048	+	+
**Genes required for the tetrahydromethanopterin (H4MPT) pathway to convert formaldehyde to formate**				
orf22	Orf22, involved in biosynthesis of tetrahydromethanopterin	MPC4_10080	+	+
orf21	Orf21, involved in biosynthesis of tetrahydromethanopterin	MPC4_10081	+	+
orf20	Orf20, involved in biosynthesis of tetrahydromethanopterin	MPC4_10082	+	+
orf19	Orf19, involved in biosynthesis of tetrahydromethanopterin	MPC4_10083	+	+
orf9	Orf9, involved in biosynthesis of tetrahydromethanopterin	MPC4_10084	+	+
orf17	Conserved HisA-related (histidine biosynthesis) protein (Orf17) involved in tetrahydromethanopterin-dependent formaldehyde oxidation	MPC4_10085	+	+
fae3	Formaldehyde-activating enzyme	MPC4_10086	+	+
orf7	Orf7, involved in tetrahydromethanopterin-dependent formaldehyde oxidation	MPC4_10087	+	+
orf5	Orf5, Conserve protein involved in biosynthesis of tetrahydromethanopterin	MPC4_10088	+	+
mch	Methenyltetrahydromethanopterin cyclohydrolase	MPC4_10089	+	+
orfY	OrfY, involved in tetrahydromethanopterin-dependent formaldehyde oxidation	MPC4_10090	+	+
mtdB	NAD(P)-dependent methylenetetrahydromethanopterin dehydrogenase	MPC4_10091	+	+
mptG	Ribofuranosylaminobenzene 5'-phosphate (RFAP) synthase	MPC4_10092	+	+
fhcC	Formyltransferase/hydrolase complex Fhc subunit C	MPC4_10072	+	+
fhcD	Formyltransferase/hydrolase complex Fhc subunit D	MPC4_10073	+	+
fhcA	Formyltransferase/hydrolase complex Fhc subunit A	MPC4_10074	+	+

fhcB	Formyltransferase/hydrolase complex Fhc subunit B	MPC4_10075	+	+
**Genes involved in the oxidation of formate to CO_2 and to produce energy**				
fdh2C	NAD-dependent formate dehydrogenase, molybdenum containing, gamma subunit	MPC4_10479	+	+
fdh2B	NAD-dependent formate dehydrogenase, molybdenum containing, beta subunit	MPC4_10480	+	+
fdh2A	NAD-linked formate dehydrogenase, molybdenum containing, alpha subunit	MPC4_10481	+	+
un-named	Accessory protein for molybdenum containing NAD-linked formate dehydrogenase	MPC4_10482	+	+
fdh2D	NAD-linked formate dehydrogenase, molybdenum containing, delta subunit	MPC4_10483	+	+

Table S4. Primers used for PCR amplification in this study.

Primer	gene	Sequence (5' – 3')*	Annealing temperature	Reference
**mmoXLF2**	*mmoX*	TGCGCGACGCCAARAAG	70˚C – 60 ˚C (Touchdown PCR)	[3]
**mmoXLR**		CCCAATCATCGCTGAAGGAGT		
**A189F**	*pmoA*	GGNGACTGGGACTTCTGG	62˚C – 52˚C (Touchdown PCR)	[4]
**Mb661R**		CCGGMGCAACGTCYTTACC		
**27F**	16S rRNA	AGAGTTTGATCMTGGCTCAG	55˚C	[5]
**1492R**		TACGGYTACCTTGTTACGACTT		
**341F**	16S rRNA	CCTACGGGNGGCWGCAG	55˚C	[6]
**785R**		GACTACHVGGGTATCTAATCC		
**1003F**	*mxaF*	GCGGCACCAACTGGGGCTGGT	65˚C – 55˚C (Touchdown PCR)	[7]
**1555R**		CATGAABGGCTCCCARTCCAT		

***** Equimolar mixtures at degenerate positions: R (G,A); N (G,A,T,C); M (A,C); Y (T,C); W (A,T); H (A,C,T) and V (G,A,C)

Table S5. Characteristics of sequenced genomes of new *Methylocella* isolates.

**Genome data**	***Methylocella* sp. PC1**	***Methylocella* sp. PC4**
Genome size (bp)	4,397,060	4,54,0050
GC content (%)	61.60	61.74
N50	134,550	148,831
L50	11	10
Number of genomic objects	5,794	4,843
Number of coding sequences (CDS)	5,705	4,753
tRNA	51	52
rRNA (16S, 5S, 23S)	One each	One each
Genome completeness (CheckM)	99.69%	99.69%
Contamination (CheckM)	1.41%	0.94%
Raw reads alignment to assembly	98.71%	99.17%

Figure S1. Relative abundance (%) of dominant bacterial phyla as revealed by 16S rRNA gene sequencing of DNA from native environmental samples.

Relative abundance (%) of dominant bacterial phyla as revealed by 16S rRNA gene sequencing of DNA from native samples from Andreiasu Everlasting Fire (Andreiasu) and Pipe Creek natural gas seep (Pipe Creek). 16S rRNA gene amplicon sequence data for Andreiasu Everlasting Fire were reported in Farhan Ul Haque et al [1].

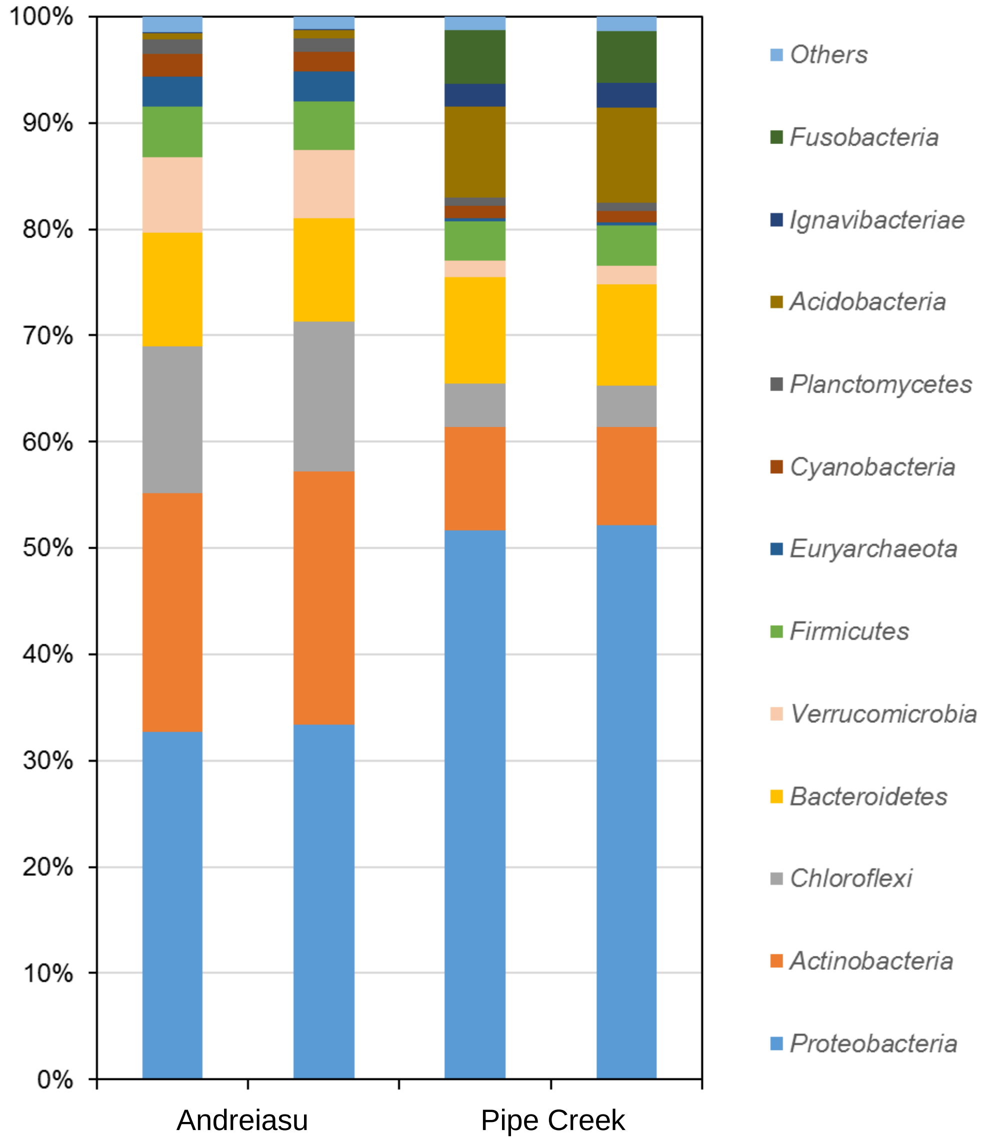

Figure S2. DNA retrieved as a function of refractive index of each fraction recovered after ultracentrifugation.

DNA retrieved as a function of refractive index of each fraction recovered from tubes following ultracentrifugation of DNA from SIP incubated samples of Andreiasu Everlasting Fire (A, B) and Pipe Creek natural gas seep (C, D). Incubations with ^{13}C-labelled CH_4 (red) and ^{12}C-labelled CH_4 (grey) were performed. Refractive indices of CsCl fractions is used as a surrogate for CsCl concentration and hence density of the solution in the gradient after ultracentrifugation. The insets in B and D represents the magnified version of the heavy fractions to show that there is increase in DNA concentration in heavy fractions of ^{13}C-labelled CH_4 incubated samples compared to those of ^{12}C-labelled CH_4 incubated samples (grey). Heavy and light fraction of time point "200 µmol C per g samples" (B and D) were used for further analyses.

A. (100 µmol C per g sample)

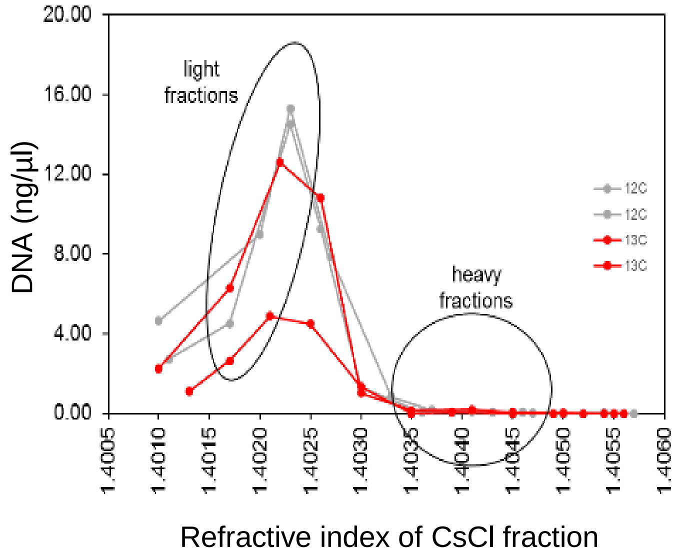

B. (200 µmol C per g sample)

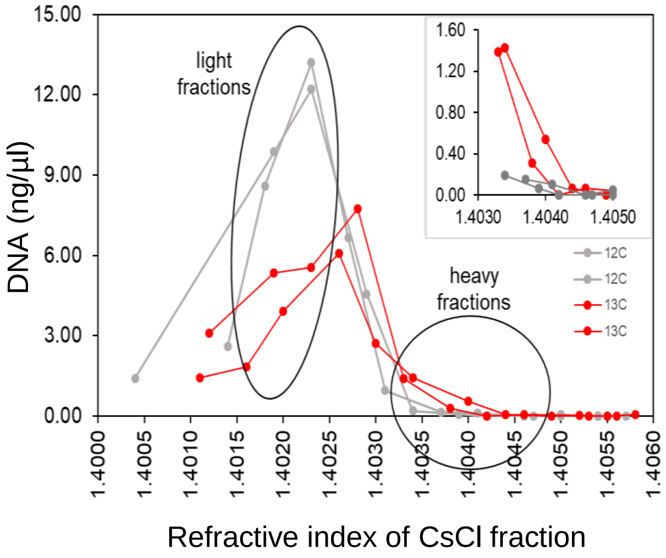

C. (100 µmol C per g sample)

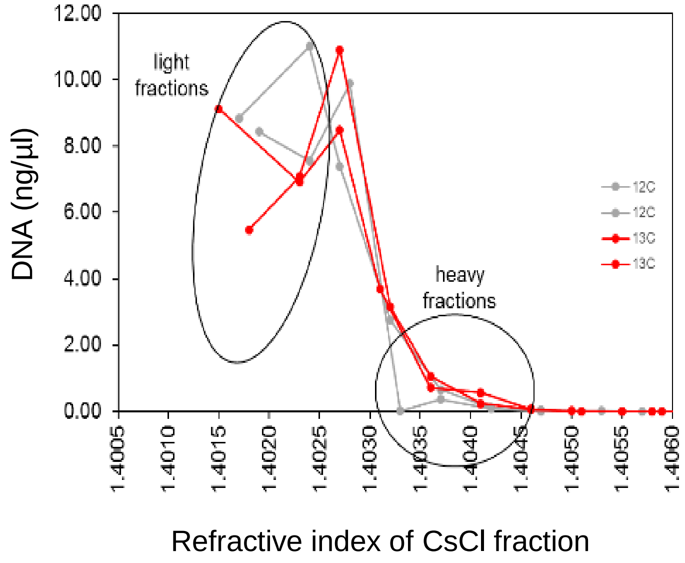

D. (200 µmol C per g sample)

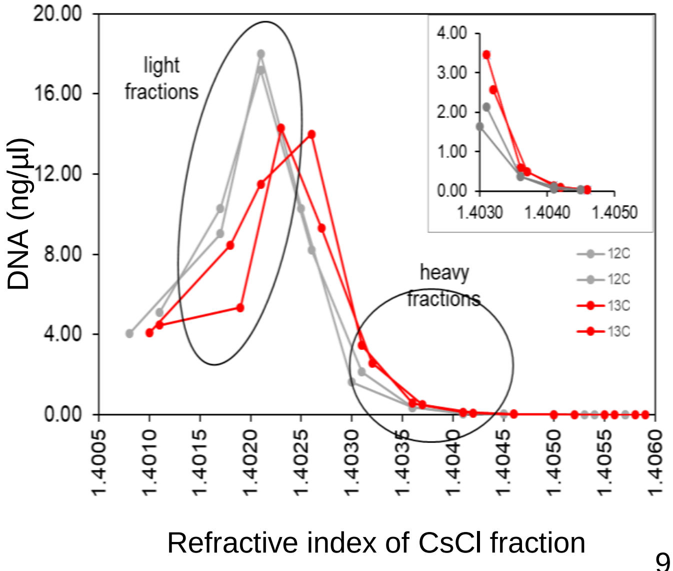

Figure S3. Growth curves of *Methylocella* sp. PC1 and PC4 under various gaseous substrates. Growth curves of *Methylocella* sp. PC1 and PC4 under various gaseous substrates. Data points (mean) and error bars (standard error of means of duplicate vials) are shown here.

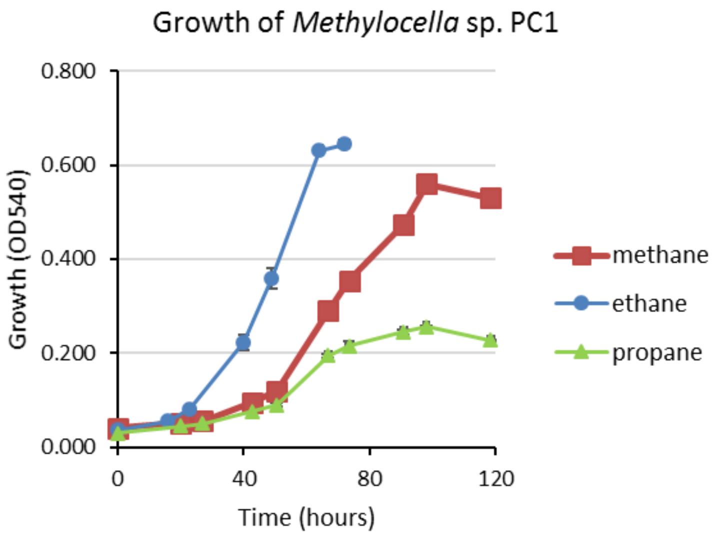

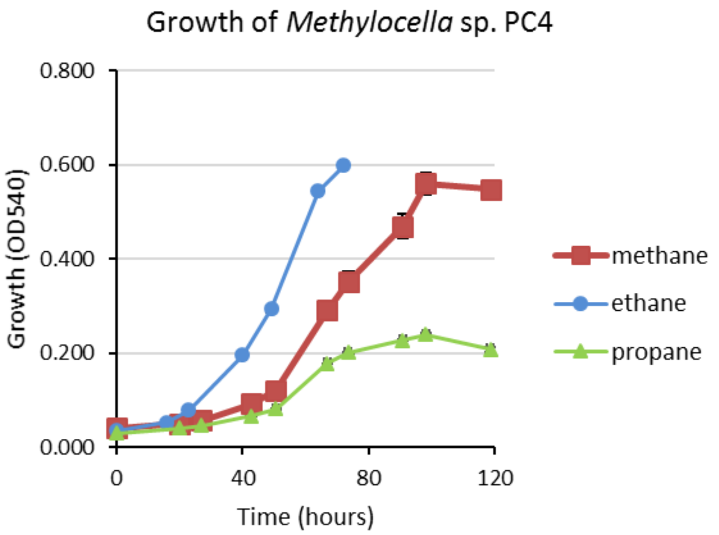

Figure S4. Phylogenetic analysis of methanol dehydrogenases from *Methylocella* PC1 and PC4 isolates.

Phylogenetic analysis of methanol dehydrogenases from *Methylocella* PC1 and PC4 isolates (bold red) along with methanol dehydrogenases from known *Methylocella* strains (bold black) and other methanotrophs based on the derived amino acid sequences of XoxF (lanthanide-dependent methanol dehydrogenase) and/or MxaF (alpha subunit of Ca-dependent methanol dehydrogenase). The tree is drawn to scale, with branch lengths measured in the number of substitutions per site. Evolutionary analyses were conducted in MEGA7 with 547 positions in the final dataset.

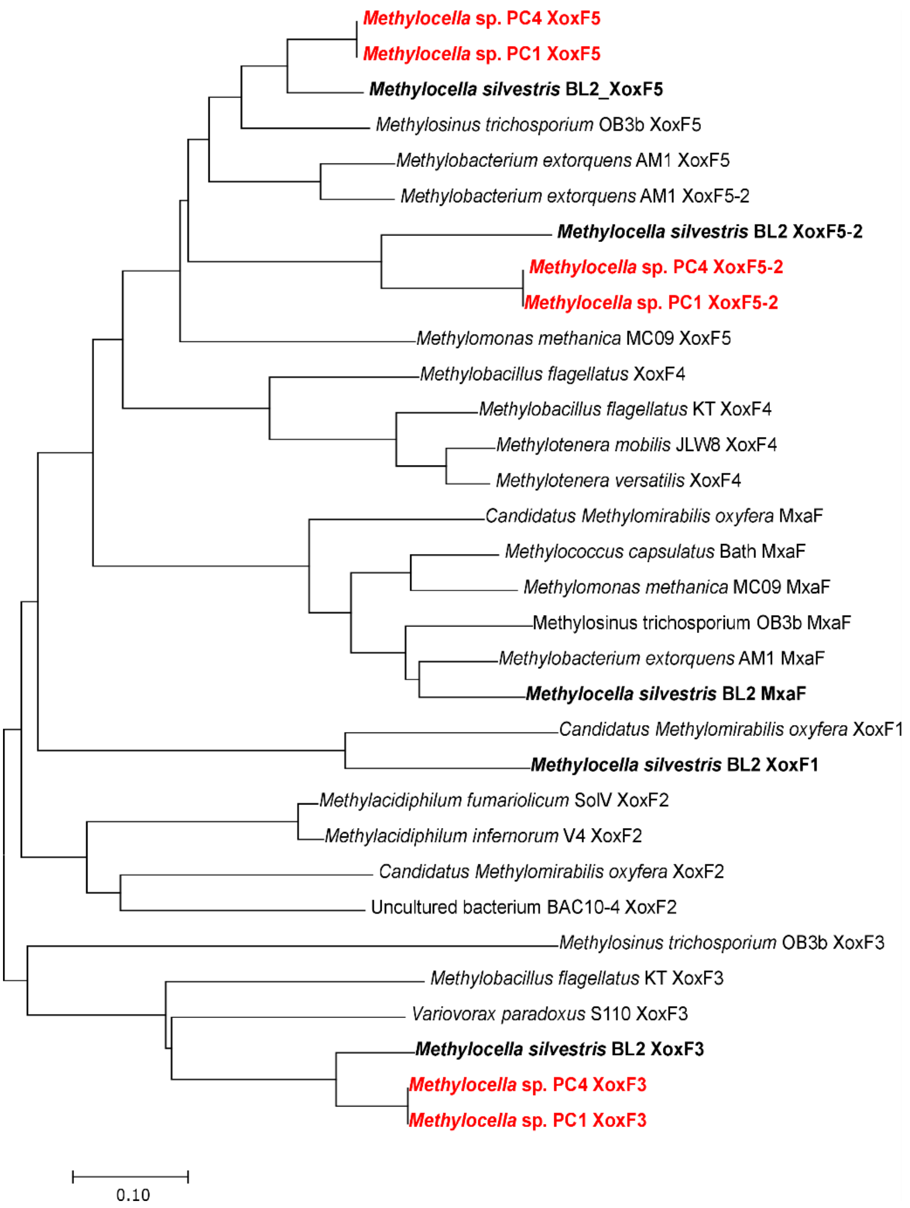

References

1. Meier-Kolthoff JP, Auch AF, Klenk H-P, Göker M: Genome sequence-based species delimitation with confidence intervals and improved distance functions. BMC Bioinformatics. 2013;14:60.
2. Meier-Kolthoff JP, Hahnke RL, Petersen J, Scheuner C, Michael V, Fiebig A, Rohde C, Rohde M, Fartmann B, Goodwin LA, et al: Complete genome sequence of DSM 30083T, the type strain (U5/41T) of Escherichia coli, and a proposal for delineating subspecies in microbial taxonomy. Standards in Genomic Sciences. 2014;9:2.
3. Farhan Ul Haque M, Crombie AT, Ensminger SA, Baciu C, Murrell JC: Facultative methanotrophs are abundant at terrestrial natural gas seeps. Microbiome. 2018;6:118.
4. Costello AM, Lidstrom ME: Molecular characterization of functional and phylogenetic genes from natural populations of methanotrophs in lake sediments. Appl Environ Microbiol. 1999;65:5066-5074.
5. Lane DJ: 16S/23S rRNA sequencing. John Wiley & Sons, New York; 1991.
6. Klindworth A, Pruesse E, Schweer T, Peplies J, Quast C, Horn M, Glöckner FO: Evaluation of general 16S ribosomal RNA gene PCR primers for classical and next-generation sequencing-based diversity studies. Nucleic Acids Res. 2013;41:e1.
7. Neufeld JD, Schäfer H, Cox MJ, Boden R, McDonald IR, Murrell JC: Stable-isotope probing implicates *Methylophaga* spp and novel *Gammaproteobacteria* in marine methanol and methylamine metabolism. The ISME Journal. 2007;1:480.
